# Supplementary material for: Outer-membrane-acting peptides and lipid II-targeting antibiotics cooperatively kill Gram-negative pathogens
Source: Commun Biol. 2021 Jan 4;4:31. doi: 10.1038/s42003-020-01511-1 (PMC7782785; doi:10.1038/s42003-020-01511-1)
Supplement: Supplementary file 2 — Supplementary Information [file 42003_2020_1511_MOESM2_ESM.pdf]

MIC in combinations (μM) and FICI		L-11	D-11	D-11R
K. pneumoniae *LMG20218*	Vancomycin	4	8	16
	Peptide	2	1	1
	FICI	**0.094**	**0.094**	**0.188**
	Nisin	0.75	0.75	3
	Peptide	1	2	2
	FICI	**0.047**	**0.078**	**0.125**
A. baumannii *LMG01041*	Vancomycin	2	2	2
	Peptide	1	1	8
	FICI	**0.070**	**0.188**	**0.188**
	Nisin	0.19	0.19	0.75
	Peptide	4	0.5	2
	FICI	**0.063**	**0.094**	**0.188**
E. aerogenes *LMG 02094*	Vancomycin	24	24	2
	Peptide	4	4	1
	FICI	**0.25**	**0.25**	**0.188**
	Nisin	4	4	2
	Peptide	8	2	4
	FICI	**0.188**	**0.188**	**0.188**
E. coli *LMG15862*	Vancomycin	4	4	8
	Peptide	1,5	0,25	0,5
	FICI	**0.188**	**0.125**	**0.188**
	Nisin	1.5	1.5	1.5
	Peptide	1.5	1	2
	FICI	**0.25**	**0.375**	**0.375**
P. aeruginosa *LMG 6395*	Vancomycin	16	32	32
	Peptide	2	4	4
	FICI	**0.25**	**0.5**	**0.5**
	Nisin	4.5	4.5	4.5
	Peptide	1	0.5	1
	FICI	**0.188**	**0.156**	**0.188**

Supplementary Table 1: MIC for the newly designed D-peptides alone in comparison with L-11, and synergistic test and FICI calculation against a Gram-negative pathogen panel.In bold synergistic combinations. For the FICI calculations twice the highest concentration tested was used in the cases where the MIC was not reached.

	**D-11**	**Vancomycin**
E. faecalis V583	64	4.13
E. faecium LMG16003	10.6	>85
S. aureus LMG8224	>85	1.37
S. pneumoniae D39	>85	0.17
S. pneumoniae R6	>85	0.34

Supplementary Table 2: MIC (µM) of D-11, and vancomycin against some Gram-positive bacteria.

	Vancomycin			D-11		
	**Alone**	**LPS**	**Mg2+**	**Alone**	**LPS**	**Mg2+**
E. coli LMG15862	64	128	256	4	>64	>64
K. pneumoniae LMG20218	128	256	>2084	32	64	>512
P. aeruginosa LMG6395	128	256	>2084	16	64	>256
A. baumannii LMG01041	32	32	256	8	128	>128
E. aerogenes LMG02094	192	384	>284	32	128	512

Supplementary Table 3: MIC value (µM) for vancomycin and D-11 alone and in the presence of LPS (1 mg/mL) or Mg^{2+} (21 mM).

Vancomycin		D-11		Vancomycin/ D-11	
**Conc.**	**MHA(%)**	**Con**	**MHA(%)**	**Con**	**MHA(%)**
31	0	50	0	22/25	0
62.5	0	100	0	45/50	0
125	0	200	0	90/100	0
250	0	400	0	180/200	0
500	0	600	0	360/400	0

	HC_{50}	GM	MHC	TI(MHC/GM)
**Vancomycin**	>500	87.7	1000	11.40
**D-11**	>200	30.75	1200	39.03

Supplementary Table 4: Hemolytic activity of vancomycin and D-11 alone and /or in combination. Conc, concentration (µM), MHA, mean hemolytic activity, HC50 is the concentration that causes 50% hemolysis of hRBCs, GM is the MIC geometric mean against all the Gram-negative strains tested, MHC is the minimal hemolytic concentration that caused 10% hemolysis of hRBCs. If there was no detectable hemolytic activity observed at 500 µM, 1000 µM was used for calculation of the therapeutic index (TI). Therapeutic index (TI) = MHC/GM. Larger values indicate greater cell specificity. TI correlates the minimal hemolytic concentration that caused 10% hemolysis MHC and the geometric mean of MIC value of all the Gram-negative strains tested (GM)

	Strains	Characteristics	References
Gram-negative strains	*Escherichia coli* LMG 15862	beta lactamase	BCCM
	E. coli ATCC BAA-2452	Clinical isolate, multidrug-resistant (MDR)	ATCC
	E. coli B1927	Clinical isolate, MDR	Fidelta Ltd collection
	E. coli ATCC 25922		ATCC
	Klebsiella pneumoniae LMG 20218	beta lactamase	BCCM
	K. pneumoniae ATCC 700603		ATCC
	K. pneumoniae ATCC BAA-2524	Clinical isolate, MDR	ATCC
	K. pneumoniae B1945	Clinical isolate, MDR	Fidelta Ltd collection
	Pseudomonas aeruginosa LMG 6395		BCCM
	P. aeruginosa ATCC 27853		ATCC
	P. aeruginosa ATCC BAA-2108	Clinical isolate, MDR	ATCC
	P. aeruginosa B1954	Clinical isolate, MDR	Fidelta Ltd collection
	P. aeruginosa PA14	Wild type strain	[1]
	Acinetobacter baumannii LMG 01041		BCCM
	A. baumannii ATCC 17978		ATCC
	A. baumannii ATCC BAA-1605	Clinical isolate, MDR	ATCC
	A. baumannii B2026	Clinical isolate, MDR	Fidelta Ltd collection
	Enterobacter aerogenes LMG 02094		BCCM
Gram-positive strains	*Enterococcus faecalis* V583	Vancomycin resitance	[2]
	E. faecium LMG 16003	Vancomycin resitance	BCCM
	Staphylococcus aureus LMG8224		BCCM
	Streptococcus pneumoniae D39		[3]
	S. pneumoniae ATCC BAA-255/ R6	No polysaccharide capsule	ATCC

Supplementary Table 5: Strains used in this work. BCCM, Belgian Coordinated Collections of Microorganism. ATCC, American Type Culture Collection. Fidelta Ltd, Zagreb, Croatia.

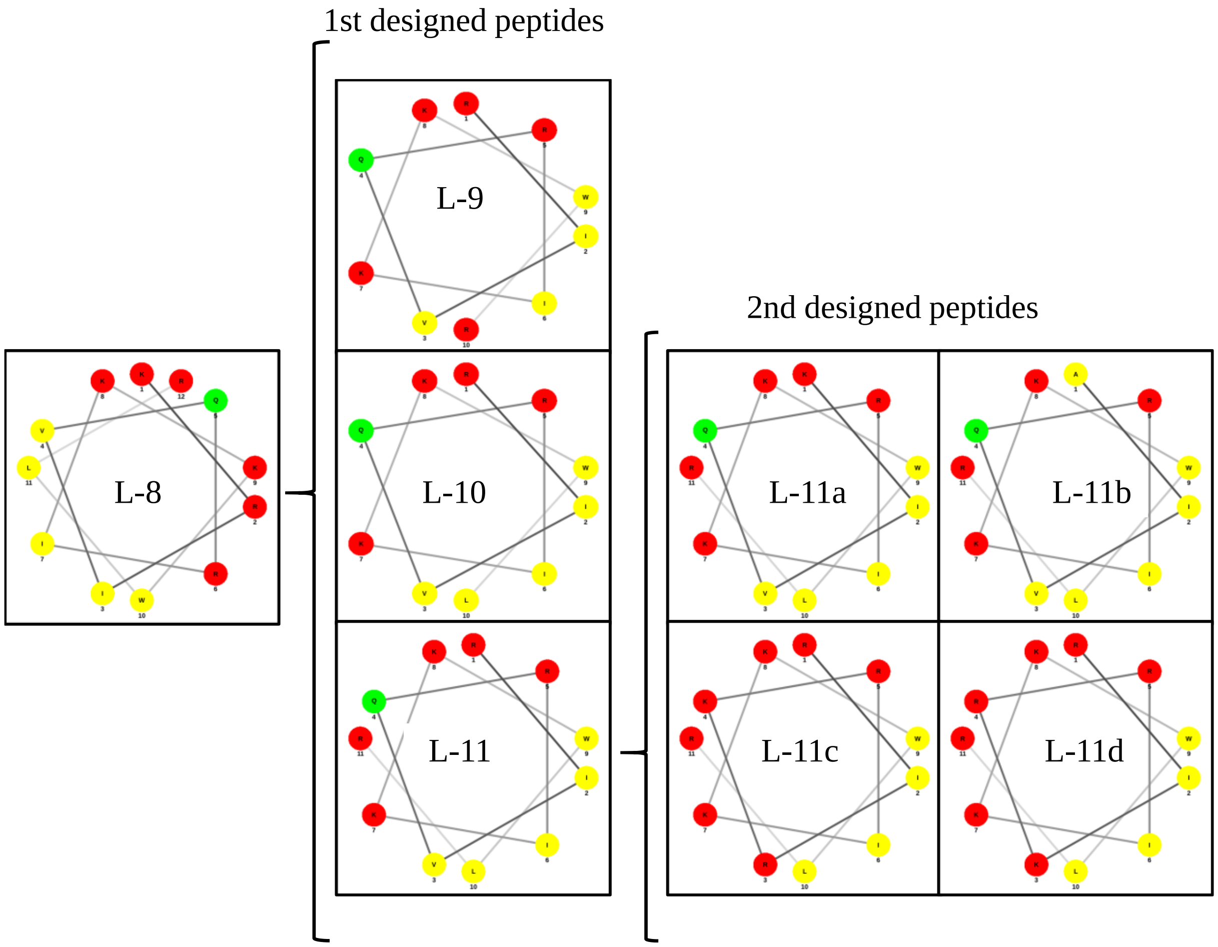

Supplementary Figure 1: Helical wheel display and sequence of the L-8 peptide derivatives.

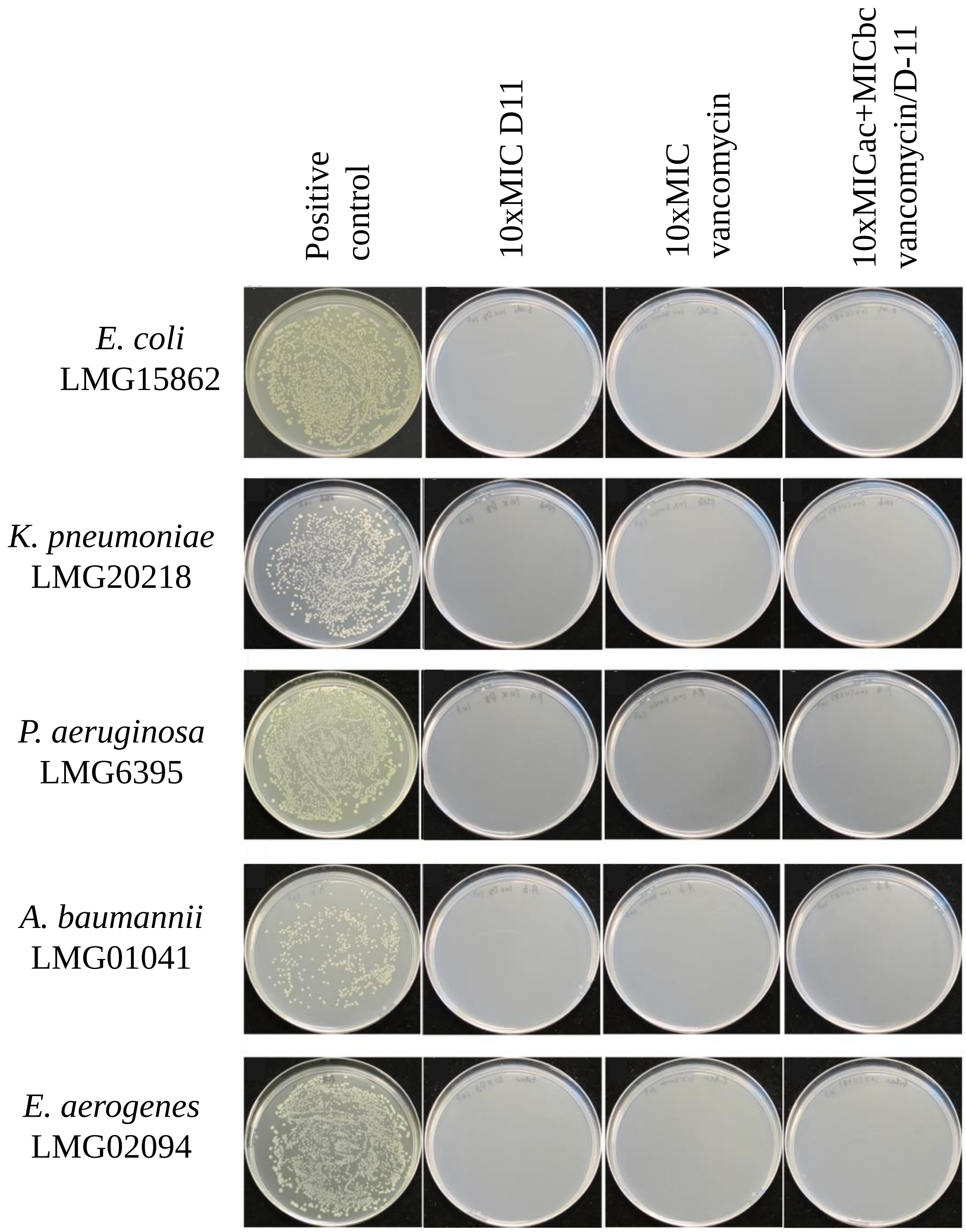

Supplementary Figure 2: Determination of viable cells after treatment with 10x vancomycin, D-11 and the combinations thereof.

Supplementary references.

1. Liberati NT, Urbach JM, Miyata S, Lee DG, Drenkard E, Wu G, Villanueva J, Wei T, Ausubel FM. 2006. An ordered, nonredundant library of *Pseudomonas aeruginosa* strain PA14 transposon insertion mutants. Proc Natl Acad Sci U S A 103:2833–2838.

2. Sahm DF, Kissinger J, Gilmore MS, Murray PR, Mulder R, Solliday J, Clarke B. 1989. In vitro susceptibility studies of vancomycin-resistant *Enterococcus faecalis*. Antimicrob Agents Chemother 33:1588–1591.

3. Lanie JA, Ng W-L, Kazmierczak KM, Andrzejewski TM, Davidsen TM, Wayne KJ, Tettelin H, Glass JI, Winkler ME. 2007. Genome sequence of Avery's virulent serotype 2 strain D39 of *Streptococcus pneumoniae* and comparison with that of unencapsulated laboratory strain R6. J Bacteriol 189:38–51.
