# Supplementary material for: Outer-membrane-acting peptides and lipid II-targeting antibiotics cooperatively kill Gram-negative pathogens
Source: Commun Biol. 2021 Jan 4;4:31. doi: 10.1038/s42003-020-01511-1 (PMC7782785; doi:10.1038/s42003-020-01511-1)
Supplement: Supplementary file 5 — Reporting Summary [file 42003_2020_1511_MOESM5_ESM.pdf]

nature research

Corresponding author(s): Oscar P. Kuipers

Last updated by author(s): Oct 23, 2020

Reporting Summary

Nature Research wishes to improve the reproducibility of the work that we publish. This form provides structure for consistency and transparency in reporting. For further information on Nature Research policies, see our Editorial Policies and the Editorial Policy Checklist.

Statistics

For all statistical analyses, confirm that the following items are present in the figure legend, table legend, main text, or Methods section.

n/a	Confirmed	
[x]	[ ]	The exact sample size (*n*) for each experimental group/condition, given as a discrete number and unit of measurement
[x]	[ ]	A statement on whether measurements were taken from distinct samples or whether the same sample was measured repeatedly
[x]	[ ]	The statistical test(s) used AND whether they are one- or two-sided<br>*Only common tests should be described solely by name; describe more complex techniques in the Methods section.*
[x]	[ ]	A description of all covariates tested
[x]	[ ]	A description of any assumptions or corrections, such as tests of normality and adjustment for multiple comparisons
[x]	[ ]	A full description of the statistical parameters including central tendency (e.g. means) or other basic estimates (e.g. regression coefficient) AND variation (e.g. standard deviation) or associated estimates of uncertainty (e.g. confidence intervals)
[x]	[ ]	For null hypothesis testing, the test statistic (e.g. *F*, *t*, *r*) with confidence intervals, effect sizes, degrees of freedom and *P* value noted<br>*Give P values as exact values whenever suitable.*
[x]	[ ]	For Bayesian analysis, information on the choice of priors and Markov chain Monte Carlo settings
[x]	[ ]	For hierarchical and complex designs, identification of the appropriate level for tests and full reporting of outcomes
[x]	[ ]	Estimates of effect sizes (e.g. Cohen's *d*, Pearson's *r*), indicating how they were calculated

Our web collection on statistics for biologists contains articles on many of the points above.

Software and code

Policy information about availability of computer code

Data collection	Tecan Infinity 200PRO, software i-control TM
Data analysis	prism 8.0, Graphpad, https://www.graphpad.com/scientific-software/prism/ and Microsoft Excel.

For manuscripts utilizing custom algorithms or software that are central to the research but not yet described in published literature, software must be made available to editors and reviewers. We strongly encourage code deposition in a community repository (e.g. GitHub). See the Nature Research guidelines for submitting code & software for further information.

Data

Policy information about availability of data

All manuscripts must include a data availability statement. This statement should provide the following information, where applicable:

- Accession codes, unique identifiers, or web links for publicly available datasets
- A list of figures that have associated raw data
- A description of any restrictions on data availability

A list of figures and tables as well as supplemental data are provide into the manuscript. The raw data for these tables and figures are available at Molecular Genetics Department (University of Groningen) o.p.kuipers@rug.nl.

Field-specific reporting

Life sciences study design

All studies must disclose on these points even when the disclosure is negative.

Sample size	Initially, 17 peptides in combination with 2 antimicrobials against 5 Gram-negative strains
Data exclusions	Data obtained during the optimization of methodologies have been not included.
Replication	At least three independent experiment for each assay were performed.In the case of mice experiment groups of 5 mice per treatment were used.
Randomization	Not applicable for this work
Blinding	No applicable

Reporting for specific materials, systems and methods

We require information from authors about some types of materials, experimental systems and methods used in many studies. Here, indicate whether each material, system or method listed is relevant to your study. If you are not sure if a list item applies to your research, read the appropriate section before selecting a response.

Materials & experimental systems

n/a	Involved in the study	
☒	☐	Antibodies
☐	☒	Eukaryotic cell lines
☒	☐	Palaeontology and archaeology
☐	☒	Animals and other organisms
☒	☐	Human research participants
☒	☐	Clinical data
☒	☐	Dual use research of concern

Methods

n/a	Involved in the study	
☒	☐	ChIP-seq
☒	☐	Flow cytometry
☒	☐	MRI-based neuroimaging

Eukaryotic cell lines

Policy information about cell lines

Cell line source(s)	Cell lines assays in this work were performed by the company Fidelta Ltd(Zagred, Croatia) involved in drug discovery services. Humans blood was obtained from the Sanquin (certified Dutch organization responsible for meeting the need in healthcare for blood and blood products, https://www.sanquin.nl/)
Authentication	Human blood was authenticated and certified by the Sanquin (The Netherlands) and the cell lines by Fidelta.
Mycoplasma contamination	Negative for Mycoplasma
Commonly misidentified lines (See ICLAC register)	no applicable

Animals and other organisms

Policy information about studies involving animals; ARRIVE guidelines recommended for reporting animal research

Laboratory animals	mice BALB/c
Wild animals	No applicable
Field-collected samples	No applicable
Ethics oversight	All animal experiments complied with US and Chinese national guidelines for the use of animals in research. The protocol was approved by the Animal Care and Use Committee of the College of Life Sciences, Nankai University (permission number NK-04-2012).

Note that full information on the approval of the study protocol must also be provided in the manuscript.
